# Identification and validation of differentially expressed chromatin regulators for diagnosis of aortic dissection using integrated bioinformatics analysis and machine-learning algorithms

**DOI:** 10.3389/fgene.2022.950613

**Published:** 2022-08-11

**Authors:** Chunjiang Liu, Yufei Zhou, Di Zhao, Luchen Yu, Yue Zhou, Miaojun Xu, Liming Tang

**Affiliations:** ^1^ Department of General Surgery, Vascular Surgery Division, Shaoxing People’s Hospital (Shaoxing Hospital of Zhejiang University), Shaoxing, China; ^2^ Department of Cardiology, Shanghai Institute of Cardiovascular Diseases, Zhongshan Hospital and Institutes of Biomedical Sciences, Fudan University, Shanghai, China; ^3^ Case Western Reserve University, Cleveland, OH, United States

**Keywords:** aortic dissection, chromatin regulator, diagnosis, bioinformatics analysis, machine learning

## Abstract

**Background:** Aortic dissection (AD) is a life-threatening disease. Chromatin regulators (CRs) are indispensable epigenetic regulators. We aimed to identify differentially expressed chromatin regulators (DECRs) for AD diagnosis.

**Methods:** We downloaded the GSE52093 and GSE190635 datasets from the Gene Expression Omnibus database. Following the merging and processing of datasets, bioinformatics analysis was applied to select candidate DECRs for AD diagnosis: CRs exertion; DECR identification using the “Limma” package; analyses of enrichment of function and signaling pathways; construction of protein–protein interaction (PPI) networks; application of machine-learning algorithms; evaluation of receiver operating characteristic (ROC) curves. GSE98770 served as the validation dataset to filter DECRs. Moreover, we collected peripheral-blood samples to further validate expression of DECRs by real-time reverse transcription-quantitative polymerase chain reaction (RT-qPCR). Finally, a nomogram was built for clinical use.

**Results:** A total of 841 CRs were extracted from the merged dataset. Analyses of functional enrichment of 23 DECRs identified using Limma showed that DECRs were enriched mainly in epigenetic-regulation processes. From the PPI network, 17 DECRs were selected as node DECRs. After machine-learning calculations, eight DECRs were chosen from the intersection of 13 DECRs identified using support vector machine recursive feature elimination (SVM-RFE) and the top-10 DECRs selected using random forest. DECR expression between the control group and AD group were considerably different. Moreover, the area under the ROC curve (AUC) of each DECR was >0.75, and four DECRs (tumor protein 53 (TP53), chromobox protein homolog 7 (CBX7), Janus kinase 2 (JAK2) and cyclin-dependent kinase 5 (CDK5)) were selected as candidate biomarkers after validation using the external dataset and clinical samples. Furthermore, a nomogram with robust diagnostic value was established (AUC = 0.960).

**Conclusion:** TP53, CBX7, JAK2, and CDK5 might serve as diagnostic DECRs for AD diagnosis. These DECRs were enriched predominantly in regulating epigenetic processes.

## 1 Introduction

Aortic dissection (AD) is an acute syndrome characterized by a tear in the inner layer of the aorta, and culminates in separation of the aortic wall (Erbel et al., 2014). Usually, AD causes severe chest pain and is life-threatening because it can result in aortic rupture and acute hypoperfusion of multiple organs (Juraszek et al., 2021). According to data from the Oxford Vascular Study, the annual incidence of AD has been estimated to be six per 100,000 people (Howard et al., 2013; Report on Cardiovascular Health and, 2021). AD is a rare syndrome, but 50% of sufferers will die in the first 48 h if surgical intervention is not provided (Helder et al., 2020). Computed tomography angiography (CTA) plays an important part in AD diagnosis, but is costly and time-consuming. Thus, rapid identification of diagnostic biomarkers for AD is essential. Multiple studies have identified the indispensable role of epigenetic mechanisms in the triggering of AD, such as post-translational modification, RNA methylation, DNA methylation, and micro (mi)RNAs (Yan and Marsden, 2015; Zarzour et al., 2019). Epigenetic alterations are actuated mainly by chromatin regulators (CRs).

In general, CRs are divided into three types based on their epigenetic regulatory roles: DNA methylators, histone modifiers, and chromatin remodelers (Lu et al., 2018). Studies have shown that abnormal expression of CRs is related to different vascular diseases. Ubiquitin like with PHD and ring finger domains 1 (UHRF-1) is an important regulatory protein that maintains the methylation of DNA and histone. UHRF-1 can directly restrict expression of the promoters of cell-cycle repressor genes and expression of key cell-promoting genes to promote the phenotype of vascular smooth muscle cells (VSMCs) during the development of aortic disease (Elia et al., 2018). Galan et al. (Galán et al., 2016) found that histone deacetylases I and IIa are overexpressed in abdominal aortic aneurysm (AAA) tissues, and are localized mainly in macrophages in the aortic wall. In mouse models, inhibitors of histone deacetylases I and IIa can reduce: 1) the risk of AAA; 2) the inflammatory response of macrophages; 3) the release of proinflammatory factors. Enhancer of zeste homolog 2 (EZH2) is a methyltransferase for the dimethylation and trimethylation of H3K27. EZH2 expression has been found to be downregulated in the aortic wall of AD patients compared with that of a control group. Moreover, EZH2 prevents the autophagic death of VSMCs by inhibiting expression of ATG5 and ATG7, and negatively regulating autophagosome formation through the MEK1-ERK1/2 signaling pathway (Li et al., 2018). CRs are also involved in inflammation (Marazzi et al., 2018), apoptosis (Krivtsov et al., 2019) and autophagy (Li et al., 2021), which take part in AD pathogenesis.

Deeper understanding of CRs is essential for the progression, diagnosis, and treatment of AD, as well as paving the way for further study. However, few scholars have investigated the connection between CRs and AD in depth. Studies based on bioinformatics analysis of CRs are scarce. Here, we aimed to identify differentially expressed chromatin regulators (DECRs) for AD diagnosis and provide a nomogram for clinical use.

## 2 Methods

### 2.1 Microarray data

**FIGURE 1:**
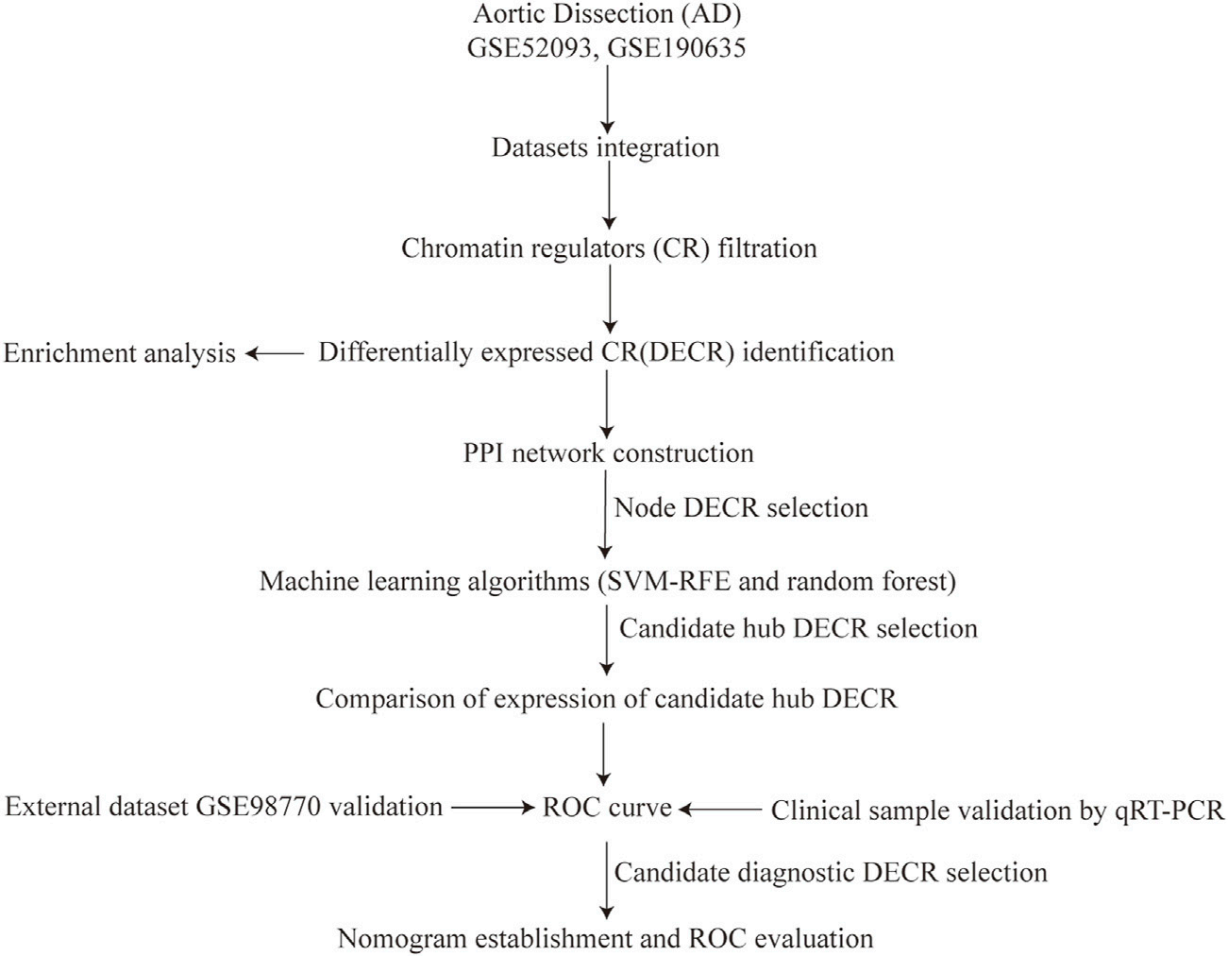
Study flowchart. Abbreviations AD, aortic dissection; CR, chromatin regulators; SVM-RFE, support vector machine-recursive feature elimination; ROC, receiver operating characteristic.

Figure 1 depicts the flowchart for the present study. Datasets were downloaded from the Gene Expression Omnibus (GEO) database (www.ncbi.nlm.nih.gov/geo/) (Barrett et al., 2013). The search strategy was (aortic dissection) AND “Homo sapiens” [porgn:__txid9606] AND “Series” AND “Expression profiling by array.” Five datasets were observed, and three of them covered our aims: GSE52093, GSE190635 and GSE98770.

GSE52093 was developed from Illumina HumanHT-12 V4.0 expression beadchips (platform: GPL10558). It comprises aorta samples from five controls and seven AD patients. GSE190635 is a new dataset generated from the Affymetrix Human Genome U133 Plus 2.0 Array (GPL570). It has not been used before, and contains eight aorta samples (four from controls and four from AD cases). GSE98770 (Kimura et al., 2017) provides expression data for messenger (m)RNAs and miRNAs. mRNA data comprises aorta samples from five controls and six AD patients (GPL14550).

### 2.2 Data processing

After downloading the raw datasets, we preprocessed the data using “affy” in R (R Institute for Statistical Computing, Vienna, Austria) to carry out background calibration, normalization, and log_2_ transformation. If multiple probes corresponded to the same gene, the median expression was calculated. After merging GSE52093 and GSE190635 datasets, the R “surrogate variable analysis” (SVA) package from Bioconductor (www.bioconductor.org/) was utilized to eliminate batch effects and other unwanted variations between the two datasets (Leek et al., 2012). GSE98770 was used as the validation dataset.

### 2.3 CR extraction and DECR identification

CR was first reported by Lu et al. (2018). We retrieved the complete CR table (870 CRs) from their study. Then, we used Perl (www.perl.org/) to extract all CRs in the merged dataset of AD. DECRs were identified using the “Limma” package in R (Ritchie et al., 2015) and visualized *via* heatmaps and volcano plots. The criteria for identifying DECRs between control samples and AD samples were |fold change| >1.5 and *p* < 0.05.

### 2.4 Analyses of functional enrichment

Gene Ontology (GO; http://geneontology.org/) and the Kyoto Encyclopedia of Genes and Genomes (KEGG; www.genome.jp/kegg/) are the main databases used for analyses of enrichment of function and signaling pathways, respectively. The GO database incorporates three ontologies: biological process (BP), cellular component (CC), and molecular function (MF) (The Gene Ontology Resource, 2019). KEGG is a knowledge resource that integrates protein–protein interaction (PPI) networks and pathways, genomic data, and chemical information (Hashimoto et al., 2006). Analyses of functional and signaling pathway enrichment were done using the package “clusterProfiler” within R. The SangerBox platform (http://vip.sangerbox.com/) was applied to visualize the results of enrichment analyses (Yu et al., 2012). Fold discovery rate <0.05 and *p* < 0.05 were set as filtration criteria.

### 2.5 PPI networks

To discover the interactions between proteins encoded by DECRs, PPI networks were built from the Search Tool for the Retrieval of Interacting Genes (STRING) database (Szklarczyk et al., 2021) (version 11.5; www.string-db.org) with the minimum needed interaction score set at 0.400. Cytoscape (https://cytoscape.org/) was applied to visualize images acquired from STRING (Otasek et al., 2019).

### 2.6 Machine learning

Two machine-learning methods [support vector machine-recursive feature elimination (SVM-RFE) and random forest] were used to further filter candidate DECRs for AD diagnosis. SVM-RFE is a sophisticated machine-learning method that can predict continuous variables while avoiding noticeable deviations (Ellis et al., 2014). Random forest is an ensemble-learning method for AD diagnosis which constructs a multitude of decision trees (Zhang et al., 2021a). The intersection DECRs from SVM-RFE and random forest were considered as candidate DECRs for AD diagnosis.

### 2.7 Establishment of receiver operating characteristic (ROC) curves

Expression of each DECR between the AD group and control group in the combined dataset was compared using the Student’s sample *t*-test based on mean ± standard deviation. ROC curves were established to assess the diagnostic value of each DECR in the combined dataset and validation dataset. The area under the ROC curve (AUC) and 95% confidence intervals (CIs) were calculated. AUC >0.7 was considered significant for AD diagnosis.

### 2.8 Collection of clinical samples

To further validate the DECRs we identified, we collected samples of peripheral blood from AD patients (*n* = 6) and healthy controls (*n* = 6). The protocol regarding sample collection was approved (2020-K-Y-116-01) by the Ethics Committee of Shaoxing People’s Hospital within Zhejiang University (Hangzhou, China). All volunteers provided written informed consent for their blood samples to be used for experimentation. All volunteers were recruited at Shaoxing People’s Hospital from 1st March 2021 to 1st March 2022. All AD patients were diagnosed with aortic dissection by computed tomography angiography (CTA). The inclusion criterion for healthy controls was that they did not have aortic disease according to CTA. The basic clinical characteristics of all participants is listed in Supplementary Table S1.

### 2.9 Real-time reverse transcription-quantitative polymerase chain reaction (RT-qPCR)

Expression of identified DECRs was validated further in clinical samples by RT-qPCR. RNA isolation and RT-qPCR were undertaken as described previously (Zhou et al., 2021). In brief, red blood cell (RBC) lysis buffer (catalog number: C3702; Beyotime Institute of Biotechnology, Shanghai, China) was used to split RBCs. Subsequent extraction of total RNA by TRIzol® Reagent and synthesis of complementary-DNA were carried out according to manufacturer (Takara Biotechnology, Shiga, Japan) protocols. mRNA expression was quantified using the ABI-9700 system (Applied Biosystems, Foster City, CA, United States) and normalized against the housekeeping gene β-actin by the comparative quantification method (2^−ΔΔCT^). The primers used in this study are listed in Supplementary Table S2.

### 2.10 Nomogram construction

After the third dataset and experimental validation, the “rms” package within R (Pan et al., 2021) was applied to build a nomogram based on candidate DECRs. “Points” denoted the score of candidate DECRs. “Total Points” referred to summation of all DECR scores. A ROC curve of the nomogram was also constructed to evaluate the value of the nomogram for diagnosing AD.

### 2.11 Statistical analyses

Prism 8.3.0 (GraphPad, San Diego, CA, United States) and SPSS 26.0 (IBM, Armonk, NY, United States) were employed for statistical analyses. For continuous variables, each comparison between two groups was calculated using the Student’s *t*-test. *p* < 0.05 was considered significant.

## 3 Results

### 3.1 CR extraction and DECR identification

**FIGURE 2:**
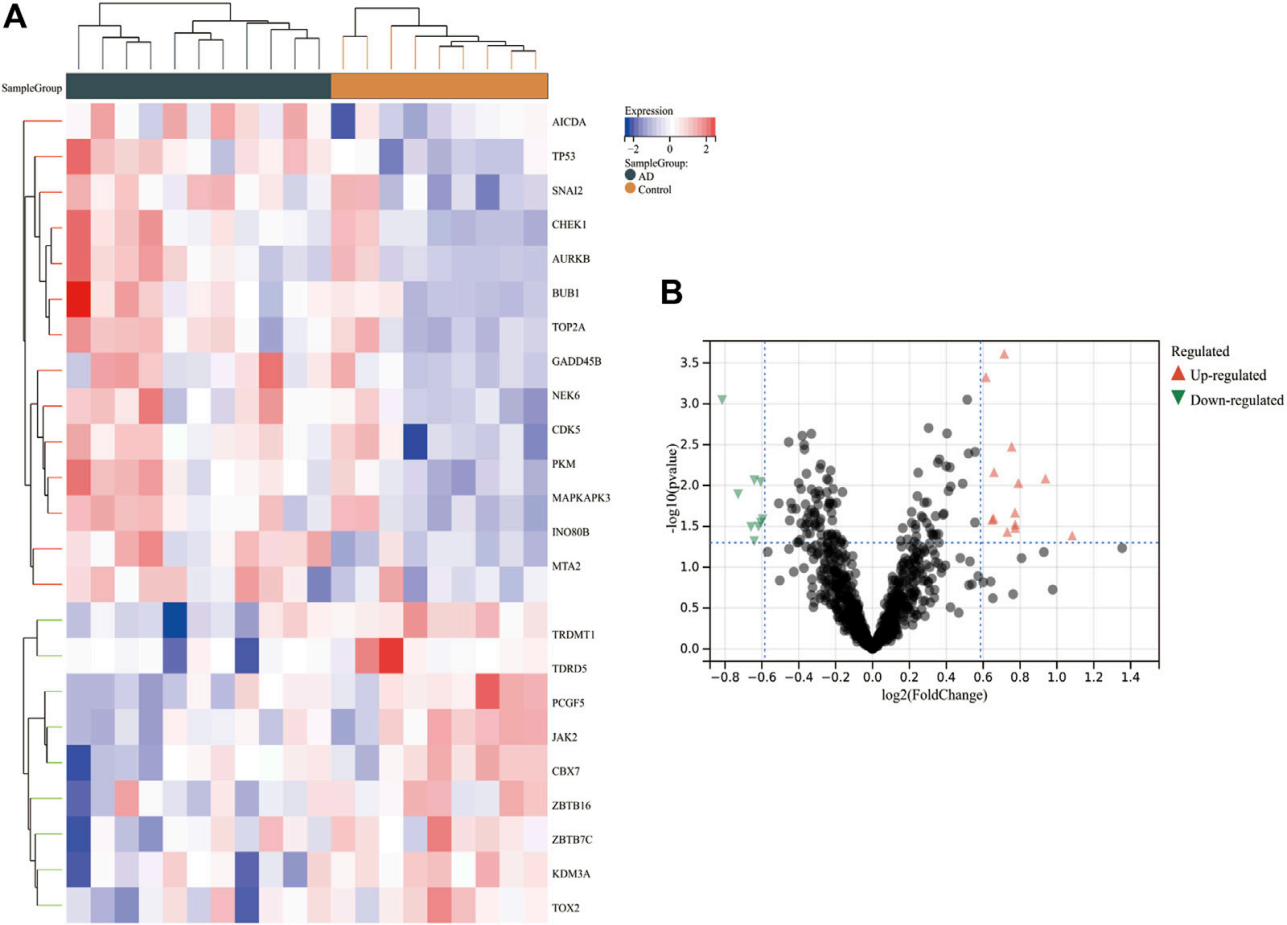
Heatmap and volcano plot of DECRs in AD. **(A)** The heatmap reveals significant DECRs between the AD group and control group. Red and blue blocks depict DECRs with upregulated expression and downregulated expression, respectively. DECRs and each sample are represented by rows and columns. **(B)** The volcano plot displays all DECRs. Red and green triangles reflect DECRs with significantly upregulated and downregulated expression, respectively. AbbreviationsDECRs, differentially expressed chromatin regulators; AD, aortic dissection.

After normalization of the combined dataset, 18,612 genes with the expression matrix were identified. A total of 841 CRs were extracted via Perl. Twenty-three DECRs were identified via Limma, of which 14 showed upregulated expression and nine had downregulated expression (Supplementary Table S3). The heatmaps and volcano plots of all DECRs are shown in Figures 2A,B.

### 3.2 Enrichment analyses of DECRs

**FIGURE 3:**
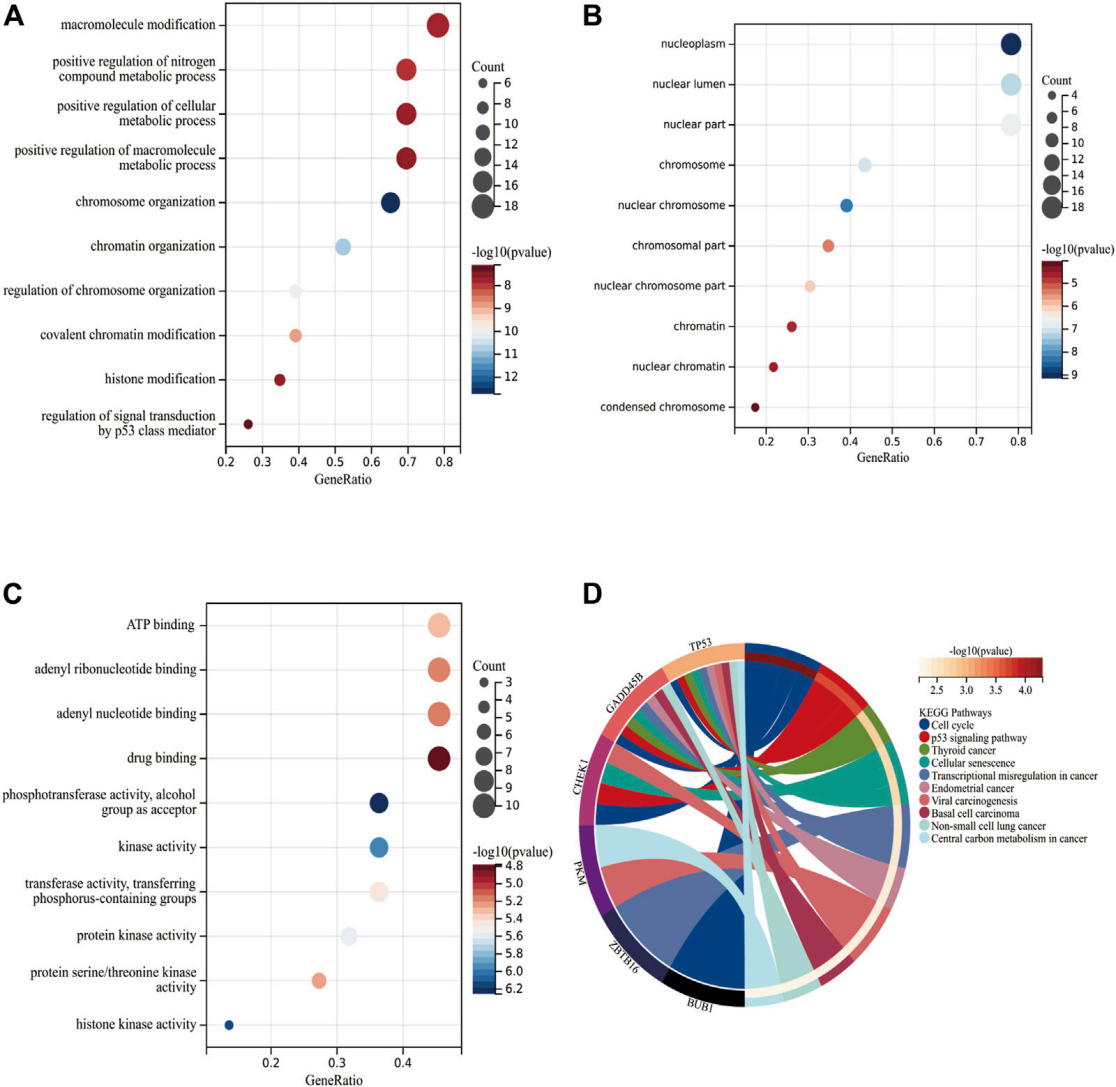
Functional enrichment of DECRs. **(A–C)** Top-10 items for biological process, cellular component, and molecular function, respectively, according to the GO database. The x-axis and y-axis represent the gene ratio and enriched items, respectively. The color and size of each circle refers to the *p*-value and gene count, respectively. **(D)** Signaling-pathway analysis of DECRs using the KEGG database. The left and right parts represent CRs and their enriched signaling pathways, respectively. AbbreviationsDECRs, differentially expressed chromatin regulators; KEGG, Kyoto Encyclopedia of Genes and Genomes.

Figures 3A–C depict the top-10 items in terms of BP, CC, and MF. With regard to BP, 23 DECRs were enriched primarily in “macromolecule modification,” “positive regulation of nitrogen compound metabolic process,” and “positive regulation of cellular metabolic process.” With respect to CC, DECRs were enriched primarily in “nucleoplasm,” “nuclear lumen,” and “nuclear part.” With regard to MF, DECRs were highly associated with “ATP binding,” “adenyl ribonucleotide binding,” and “adenyl nucleotide binding.” The KEGG database revealed that DECRs were enriched mainly in “cell cycle” and “p53 signaling pathway” (Figure 3D and Supplementary Table S4).

### 3.3 PPI networks

**FIGURE 4:**
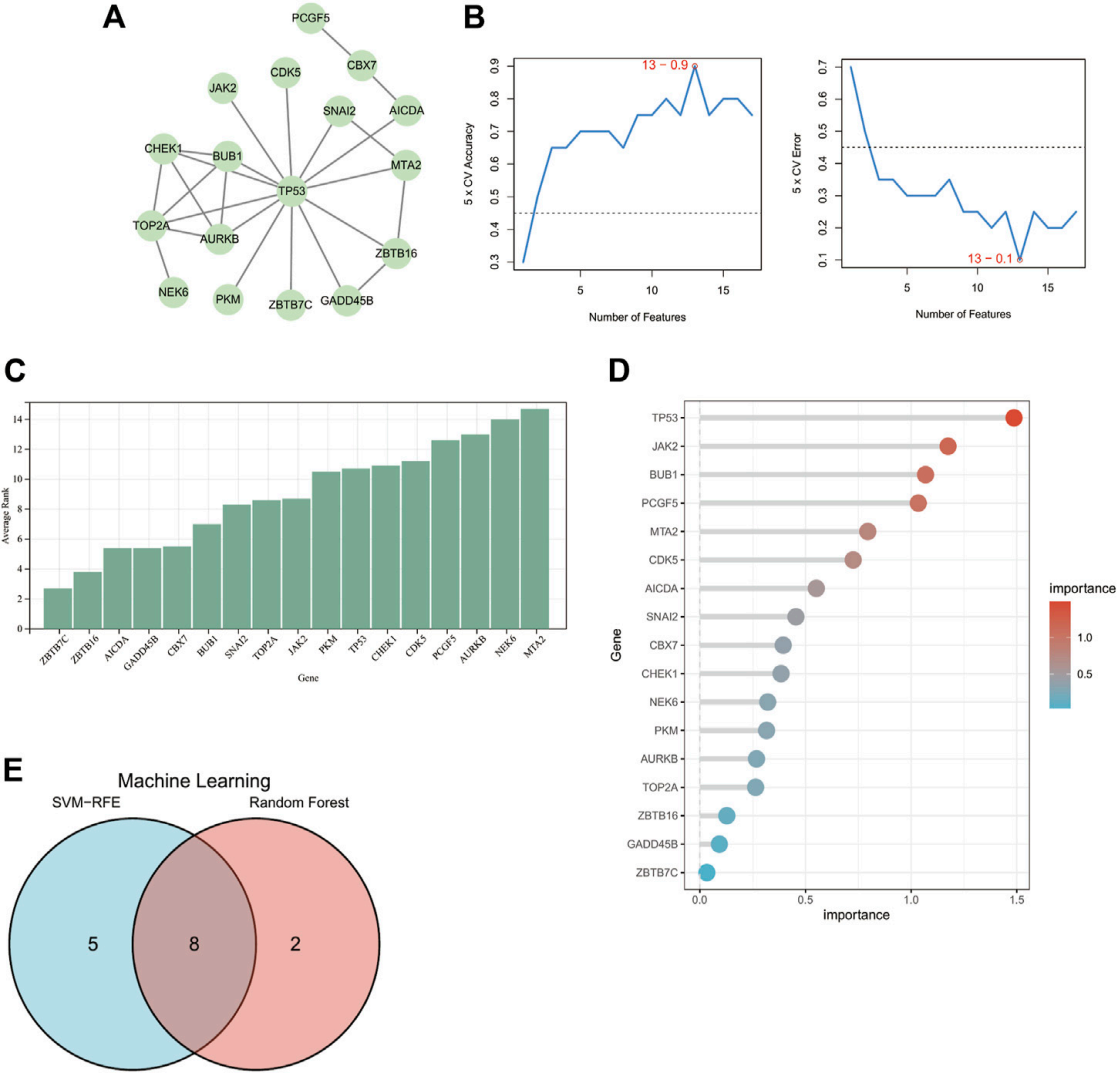
Identification of candidate DECRs for AD diagnosis. **(A)** The PPI network shows that 17 DECRs can interact with each other. DECRs devoid of interaction were eliminated. **(B)** The SVM-RFE machine-learning algorithm illustrates that, based on the 17 DECRs, the top-13 DECRs show the highest accuracy and lowest error for AD diagnosis. **(C)** DECRs were ranked based on calculation of average rank score from SVM-RFE. Genes with a lower rank denote a higher diagnostic value. **(D)** The importance score for each DECR can be visualized from the column using the random-forest algorithm. **(E)** The venn diagram shows that eight DECRs were filtered for subsequent evaluation of ROC curves. AbbreviationsDECRs, differentially expressed chromatin regulators; AD, aortic dissection; PPI, protein–protein interaction; SVM-RFE, support vector machine-recursive feature elimination; ROC, receiver operating characteristic.

To select candidate DECRs for AD diagnosis, we first constructed PPI networks to filter DECRs. Seventeen DECRs were found to interact with other DECRs in the network with 25 edges (Figure 4A). In a preliminary manner, this finding showed the potential diagnostic value of the identified DECRs, with TP53 being the most significant DECR. Six DECRs were deleted because they did not link with other DECRs.

### 3.4 Selection of candidate genes *via* machine-learning algorithms

We used two machine-learning algorithms to select candidate DECRs. Only DECRs identified from both algorithms were chosen for further evaluation of diagnostic value.

Figure 4B shows the top-13 DECRs chosen from 17 DECRs based on the highest accuracy and lowest error points using the SVM-RFE algorithm. Figure 4C depicts the average rank of 17 DECRs chosen using SVM-RFE. The top-10 DECRs were picked for their intersection with the 13 discovered DECRs from SVM-RFE based on the significance determined using random forest (Figure 4D). Eight DECRs (cyclin-dependent kinase 5 (CDK5), Janus kinase 2 (JAK2), BUB1 mitotic checkpoint serine/threonine kinase (BUB1), chromobox protein homolog 7 (CBX7), snail family transcriptional repressor 2 (SNAI2), checkpoint kinase 1 (CHEK1), tumor protein P53 (TP53), activation-induced cytidine deaminase (AICDA)) visualized from a Venn diagram (Figure 4E) were selected for evaluation of diagnostic value.

### 3.5 Assessment of diagnostic value

**FIGURE 5:**
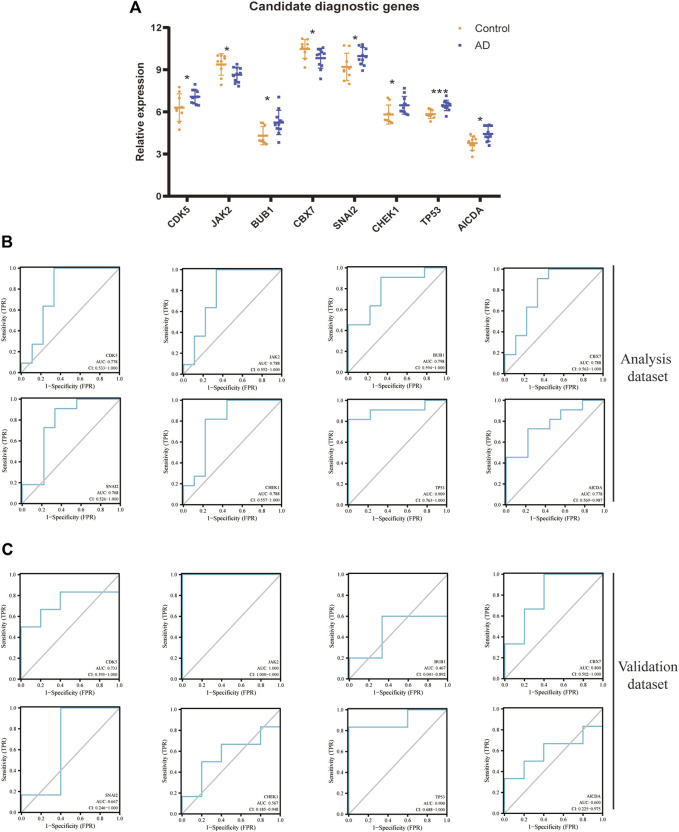
Diagnostic value of each DECR. **(A)** Statistical analyses regarding expression of eight candidate DECRs between samples from AD cases and healthy controls. Yellow and blue plots represent healthy-control and AD samples, respectively. *p < 0.05; ***p < 0.001. **(B)** The ROC curve of each candidate DECR shows the diagnostic value. The AUC and 95%CI are listed at the bottom. **(C)** The other validation dataset of AD was applied to analyze the diagnostic value revealed by the ROC curve. AbbreviationsDECR, differentially expressed chromatin regulator; AD, aortic dissection; ROC, receiver operating characteristic; AUC, area under the ROC curve; CI, confidence interval.

Figure 5A shows a comparison of expression of eight DECRs between the AD group and control group. All eight DECRs had significantly different expression, with TP53 having the greatest variation between two groups. Analyses of ROC curves for each DECR revealed AUC >0.75, which indicated strong diagnostic value (Figure 5B). Due to constraints in terms of sample size, we chose the other AD dataset, GSE98770, for additional validation. AUC >0.7 for CDK5, JAK2, CBX7, and TP53 (Figure 5C), whereas AUC <0.7 for BUB1, CHEK1, AICDA, and SNAI2.

### 3.6 Experimental validation and nomogram construction

**FIGURE 6:**
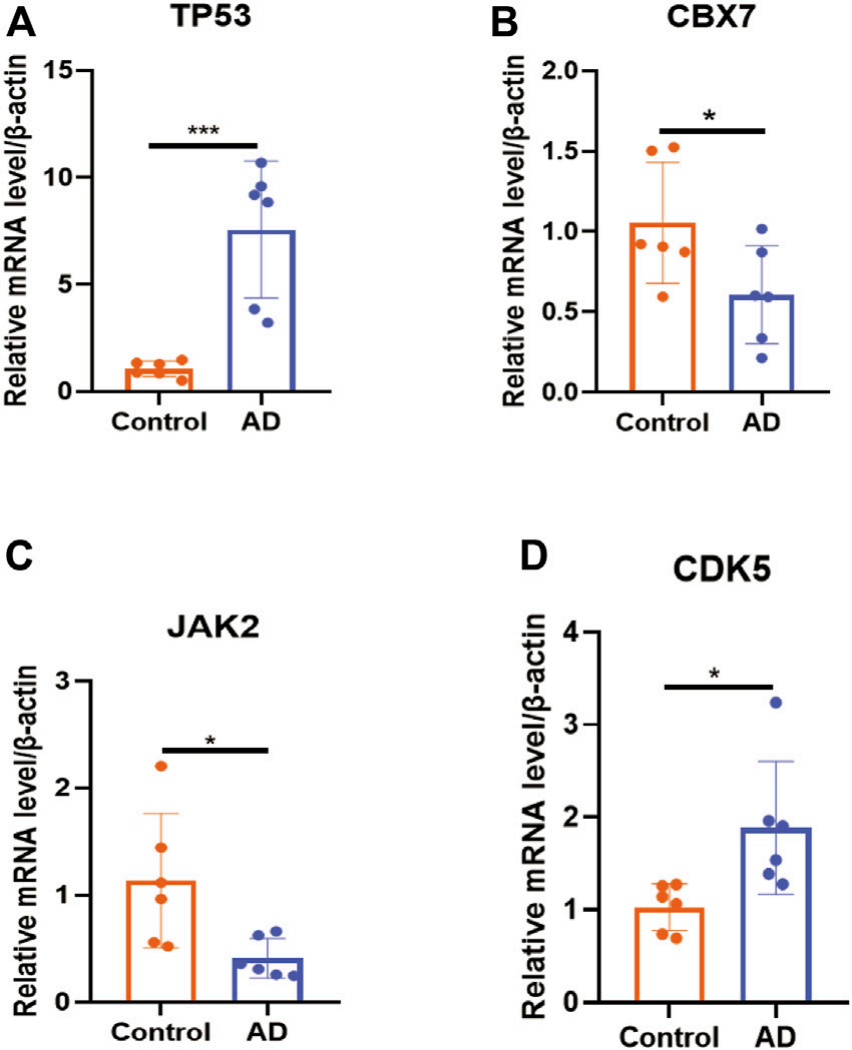
Validation of expression of each DECR by RT-qPCR. **(A–D)** Expression of DECRs (TP53, CBX7, JAK2, and CDK5) between samples from healthy controls and AD patients (*n* = 6 per group). *p < 0.05, ***p < 0.001. AbbreviationsDECR, differentially expressed chromatin regulator; RT-qPCR, reverse transcription-quantitative polymerase chain reaction; AD, aortic dissection; TP53, tumor protein P53, CBX7, chromobox protein homolog 7; JAK2, Janus kinase 2; CDK5, cyclin-dependent kinase 5.

**FIGURE 7:**
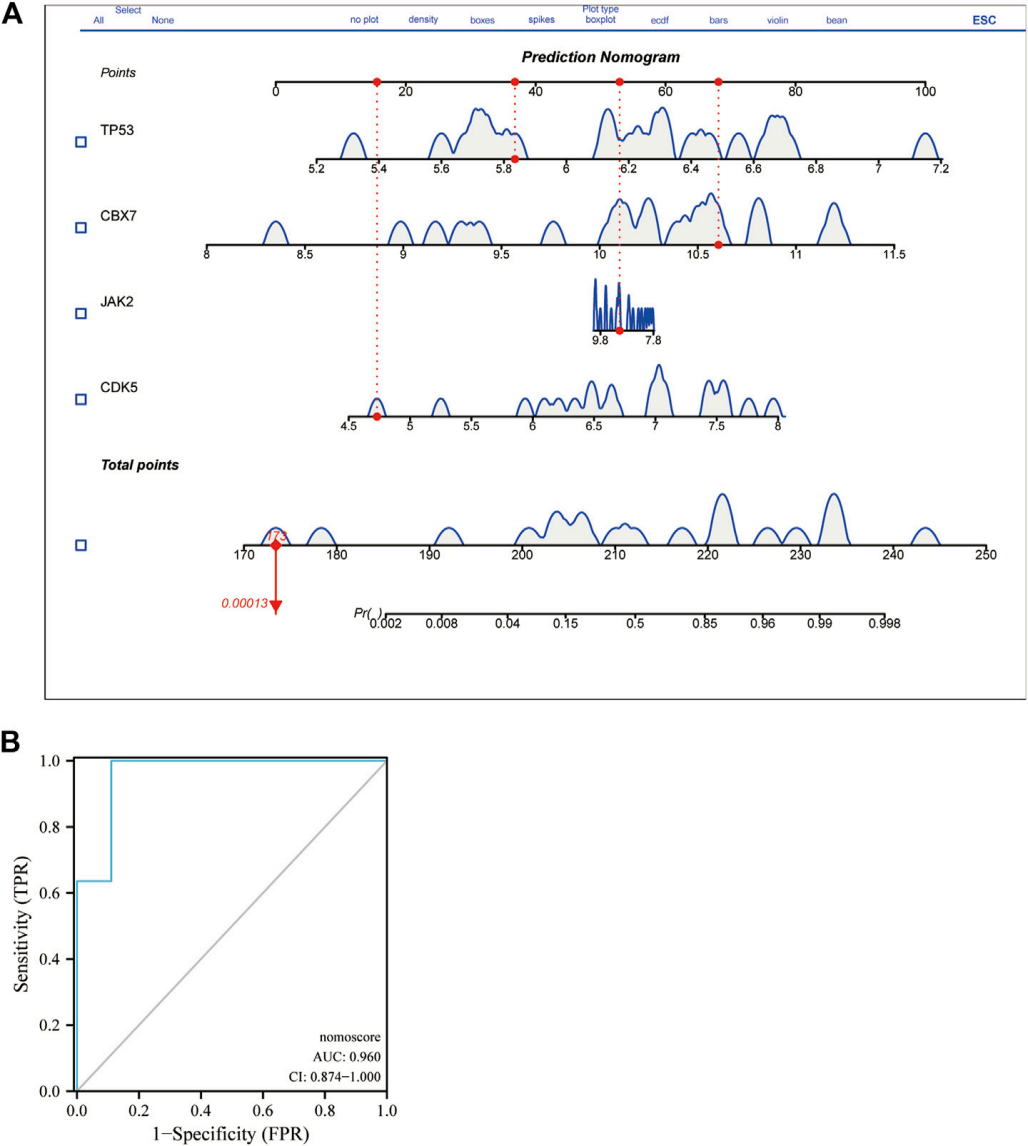
Nomogram of DECR for AD diagnosis and evaluation of diagnostic value visualized from ROC curves. **(A)** A nomogram was developed using four externally validated DECRs. Each DECR corresponds to a score on the nomogram. The final score was determined by adding the scores for each DECR. **(B)** ROC curve of the nomogram revealed DECRs with prominent diagnostic value for AD. AbbreviationsDECR; differentially expressed chromatin regulator; AD, aortic dissection; ROC, receiver operating characteristic.

Using peripheral-blood samples, we discovered that expression of CDK5 and TP53 was significantly higher in AD patients compared with that in healthy controls. Expression of CBX7 and JAK2 was downregulated in AD samples (Figure 6). These data further validated the reliability of the four DECRs we identified. A nomogram was established based on CDK5, TP53, CBX7, and JAK2 (Figure 7A), and the AUC was 0.960 (95%CI: 0.874–1.000), thereby demonstrating its high value for diagnosing AD (Figure 7B).

## 4 Discussion

Studies have focused mainly on discovering biomarkers and treatment strategies for AD. For instance, Forrer et al. (2021) found that interleukin 10 (IL-10) performed best among several proinflammatory biomarkers for AD diagnosis. In a study recruiting patients with suspected AD in the emergency setting, soluble suppression of tumorigenicity-2 showed better overall diagnostic performance than D-dimer or cardiac troponin I (Wang et al., 2018). However, studies have not investigated the value of CRs for AD diagnosis.

Here, based on bioinformatics analysis and machine-learning methods, we discovered four DECRs (TP53, CBX7, JAK2, and CDK5) and built a nomogram for AD diagnosis.

According to the GO database, the DECRs were associated primarily with epigenetic processes: “chromosome organization regulation,” “histone modification,” “RNA biosynthetic process regulation,” and “metabolic process regulation.” The DECRs were found to be engaged mainly in the “cell cycle,” “p53 signaling pathway,” and “MAPK signaling pathway” according to the KEGG database. These pathways are closely connected to vascular disorders, which implies that the DECRs may have important roles in the development and progression of AD.

Studies have unraveled only partially the role of epigenetics in the pathogenesis and progression of CRs. Bellenguez et al. discovered that expression of histone deacetylase-9 was upregulated in different VSMC types in people with a thoracic aortic aneurysm (Bellenguez et al., 2012). Rho GTPase activating protein-18 was found by Liu and colleagues to protect against formation of thoracic aortic aneurysms by inhibiting the synthetic and proinflammatory phenotypes of smooth muscle cells (Liu et al., 2017).

TP53 acts as a tumor suppressor and promotes growth arrest or apoptosis depending on physiological conditions and cell type. Through the TP53 pathway, some molecules have been found to influence neointima formation, VSMC proliferation, and apoptosis (Wu et al., 2014; Hennigs et al., 2021). Several studies have focused on TP53 and cardiovascular disorders (particularly cardiomyopathy and cardiomyocyte maturation). Chen et al. (2019) discovered that activation of the DNA damage response/TP53 pathway could lead to development of dilated cardiomyopathy. Rouhi et al. (Rouhi et al., 2022) demonstrated that the TP53 pathway was activated in human hearts with arrhythmogenic cardiomyopathy in the absence of overt cardiac failure. Shoffner et al. (Shoffner et al., 2020) discovered that inhibiting TP53 expression promoted cardiomyocyte proliferation during regeneration of zebrafish hearts. Our investigation indicated that TP53 was the most important DECR for AD diagnosis, with the highest diagnostic value in the test dataset and validation dataset. TP53 showed substantial expression in AD patients, and was involved primarily in epigenetic regulation, including histone modification and regulation of cellular metabolic processes. Hence, future research should concentrate on unraveling the exact epigenetic mechanism of TP53 in AD pathogenesis.

CBX7 is a component of a polycomb-group multiprotein PRC1-like complex. It plays a part in chromatin remodeling and histone modification. Suppression of CBX7 expression has revealed protective effects against ischemia–reperfusion injury in the kidney (Zhang et al., 2020) and brain (Zhang et al., 2021b) *via* different pathways. We showed that CBX7 expression was downregulated in AD patients, suggesting that this is a compensatory response in AD pathogenesis. JAK2 is involved in the growth, development, and differentiation of cells and histone modification. Several scholars have reported the role of JAK2 in AD pathogenesis. Ren et al. (Ren et al., 2017) found the JAK2/signal transducer and activator of transcription-3 pathway to be related to the protective effects of IL-22 in patients with AD and acute lung injury. Pan and colleagues (Pan et al., 2014) constructed a JAK2-centered interactome “hotspot” identified by an integrative network algorithm in Stanford type-A acute AD. Our results are consistent with those in other studies.

CDK5 is essential for regulation of the cell cycle. Studies on CDK5 have mainly revealed enriched regulation of neuro-homeostasis. Johana et al. (Gutiérrez-Vargas et al., 2015) found that knockdown of CDK5 expression could prevent the hippocampal degeneration and cognitive dysfunction produced by cerebral ischemia. Inhibition of CDK5 activity after hypoxia-–ischemia injury can reduce infarct size and promote functional recovery in neonatal rats (Tan et al., 2015). CDK5 expression was higher in AD patients in the present study, which showed that suppression of CDK5 expression could serve as a potential target for AD treatment.

Our study had two main drawbacks. First, despite combining two datasets and selecting a validation dataset as well as validation of clinical samples by RT-qPCR in our hospital, the sample size was limited. Second, the underlying mechanisms between DECRs and epigenetic regulation need further investigation.

## 5 Conclusion

Using a combination of bioinformatics analysis and machine-learning methods, we discovered four DECRs (CDK5, JAK2, CBX7, and TP53) that could be used for AD diagnosis. We created a nomogram with the highest diagnostic value for clinical application. All the DECRs we discovered were enriched primarily in regulating epigenetic processes, which indicated the potential utility of regulating epigenetic processes in the diagnosis and therapy of AD.

## Data Availability

The original contributions presented in the study are included in the article/Supplementary Material, further inquiries can be directed to the corresponding author.

## References

[B1] Barrett T. Wilhite S. E. Ledoux P. Evangelista C. Kim I. F. Tomashevsky M. (2013). NCBI GEO: Archive for functional genomics data sets--update. Nucleic Acids Res. 41, D991–D995. 10.1093/nar/gks1193 23193258 PMC3531084

[B2] Bellenguez C. Bevan S. Gschwendtner A. Spencer C. C. Burgess A. I. Pirinen M. (2012). Genome-wide association study identifies a variant in HDAC9 associated with large vessel ischemic stroke. Nat. Genet. 44 (3), 328–333. 10.1038/ng.1081 22306652 PMC3303115

[B3] Chen S. N. Lombardi R. Karmouch J. Tsai J. Y. Czernuszewicz G. Taylor M. R. G. (2019). DNA damage response/TP53 pathway is activated and contributes to the pathogenesis of dilated cardiomyopathy associated with LMNA (lamin A/C) mutations. Circ. Res. 124 (6), 856–873. 10.1161/CIRCRESAHA.118.314238 30696354 PMC6460911

[B4] Elia L. Kunderfranco P. Carullo P. Vacchiano M. Farina F. M. Hall I. F. (2018). UHRF1 epigenetically orchestrates smooth muscle cell plasticity in arterial disease. J. Clin. Invest. 128 (6), 2473–2486. 10.1172/JCI96121 29558369 PMC5983314

[B5] Ellis K. Kerr J. Godbole S. Lanckriet G. Wing D. Marshall S. (2014). A random forest classifier for the prediction of energy expenditure and type of physical activity from wrist and hip accelerometers. Physiol. Meas. 35 (11), 2191–2203. 10.1088/0967-3334/35/11/2191 25340969 PMC4374571

[B6] Erbel R. Aboyans V. Boileau C. Bossone E. Di Bartolomeo R. Eggebrecht H. (2014). 2014 ESC Guidelines on the diagnosis and treatment of aortic diseases: Document covering acute and chronic aortic diseases of the thoracic and abdominal aorta of the adult. The Task Force for the Diagnosis and Treatment of Aortic Diseases of the European Society of Cardiology (ESC). Eur. Heart J. 35 (41), 2873–2926. 10.1093/eurheartj/ehu281 25173340

[B7] Forrer A. Schoenrath F. Torzewski M. Schmid J. Franke U. F. W. Gobel N. (2021). Novel blood biomarkers for a diagnostic workup of acute aortic dissection. Diagnostics 11 (4), 615. 10.3390/diagnostics11040615 33808169 PMC8065878

[B8] Galán M. Varona S. Orriols M. Rodríguez J. A. Aguiló S. Dilmé J. (2016). Induction of histone deacetylases (HDACs) in human abdominal aortic aneurysm: Therapeutic potential of HDAC inhibitors. Dis. Model. Mech. 9 (5), 541–552. 10.1242/dmm.024513 26989193 PMC4892665

[B9] Gutiérrez-Vargas J. A. Múnera A. Cardona-Gómez G. P. (2015). CDK5 knockdown prevents hippocampal degeneration and cognitive dysfunction produced by cerebral ischemia. J. Cereb. Blood Flow. Metab. 35 (12), 1937–1949. 10.1038/jcbfm.2015.150 26104286 PMC4671113

[B10] Hashimoto K. Goto S. Kawano S. Aoki-Kinoshita K. F. Ueda N. Hamajima M. (2006). KEGG as a glycome informatics resource. Glycobiology 16 (5), 63R–70r. 10.1093/glycob/cwj010 16014746

[B11] Helder M. R. K. Schaff H. V. Day C. N. Pochettino A. Bagameri G. Greason K. L. (2020). Regional and temporal trends in the outcomes of repairs for acute type A aortic dissections. Ann. Thorac. Surg. 109 (1), 26–33. 10.1016/j.athoracsur.2019.06.058 31400338

[B12] Hennigs J. K. Cao A. Li C. G. Shi M. Mienert J. Miyagawa K. (2021). PPARγ-p53-Mediated vasculoregenerative program to reverse pulmonary hypertension. Circ. Res. 128 (3), 401–418. 10.1161/CIRCRESAHA.119.316339 33322916 PMC7908816

[B13] Howard D. P. Banerjee A. Fairhead J. F. Perkins J. Silver L. E. Rothwell P. M. (2013). Population-based study of incidence and outcome of acute aortic dissection and premorbid risk factor control: 10-year results from the Oxford vascular study. Circulation 127 (20), 2031–2037. 10.1161/CIRCULATIONAHA.112.000483 23599348 PMC6016737

[B14] Juraszek A. Czerny M. Rylski B. (2021). Update in aortic dissection. Trends cardiovasc. Med. 10.1016/j.tcm.2021.08.008 34411744

[B15] Kimura N. Futamura K. Arakawa M. Okada N. Emrich F. Okamura H. (2017). Gene expression profiling of acute type A aortic dissection combined with *in vitro* assessment. Eur. J. Cardiothorac. Surg. 52 (4), 810–817. 10.1093/ejcts/ezx095 28402522

[B16] Krivtsov A. V. Evans K. Gadrey J. Y. Eschle B. K. Hatton C. Uckelmann H. J. (2019). A menin-MLL inhibitor induces specific chromatin changes and eradicates disease in models of MLL-rearranged leukemia. Cancer Cell 36 (6), 660–673. e611. 10.1016/j.ccell.2019.11.001 31821784 PMC7227117

[B17] Leek J. T. Johnson W. E. Parker H. S. Jaffe A. E. Storey J. D. (2012). The sva package for removing batch effects and other unwanted variation in high-throughput experiments. Bioinformatics 28 (6), 882–883. 10.1093/bioinformatics/bts034 22257669 PMC3307112

[B18] Li Q. Ni Y. Zhang L. Jiang R. Xu J. Yang H. (2021). HIF-1α-induced expression of m6A reader YTHDF1 drives hypoxia-induced autophagy and malignancy of hepatocellular carcinoma by promoting ATG2A and ATG14 translation. Signal Transduct. Target. Ther. 6 (1), 76. 10.1038/s41392-020-00453-8 33619246 PMC7900110

[B19] Li R. Yi X. Wei X. Huo B. Guo X. Cheng C. (2018). EZH2 inhibits autophagic cell death of aortic vascular smooth muscle cells to affect aortic dissection. Cell Death Dis. 9 (2), 180. 10.1038/s41419-017-0213-2 29416002 PMC5833461

[B20] Liu R. Lo L. Lay A. J. Zhao Y. Ting K. K. Robertson E. N. (2017). ARHGAP18 protects against thoracic aortic aneurysm formation by mitigating the synthetic and proinflammatory smooth muscle cell phenotype. Circ. Res. 121 (5), 512–524. 10.1161/CIRCRESAHA.117.310692 28701309

[B21] Lu J. P. Xu J. Li J. Y. Pan T. Bai J. Wang L. Q. (2018). Facer: Comprehensive molecular and functional characterization of epigenetic chromatin regulators. Nucleic Acids Res. 46 (19), 10019–10033. 10.1093/nar/gky679 30102398 PMC6212842

[B22] Marazzi I. Greenbaum B. D. Low D. H. P. Guccione E. (2018). Chromatin dependencies in cancer and inflammation. Nat. Rev. Mol. Cell Biol. 19 (4), 245–261. 10.1038/nrm.2017.113 29184195

[B23] Otasek D. Morris J. H. Bouças J. Pico A. R. Demchak B. (2019). Cytoscape automation: Empowering workflow-based network analysis. Genome Biol. 20 (1), 185. 10.1186/s13059-019-1758-4 31477170 PMC6717989

[B24] Pan S. Wu D. Teschendorff A. E. Hong T. Wang L. Qian M. (2014). JAK2-centered interactome hotspot identified by an integrative network algorithm in acute Stanford type A aortic dissection. PLoS One 9 (2), e89406. 10.1371/journal.pone.0089406 24586754 PMC3933461

[B25] Pan X. Jin X. Wang J. Hu Q. Dai B. (2021). Placenta inflammation is closely associated with gestational diabetes mellitus. Am. J. Transl. Res. 13 (5), 4068–4079. 34149999 PMC8205654

[B26] Ren W. Wang Z. Wu Z. Hu Z. Dai F. Chang J. (2017). JAK2/STAT3 pathway was associated with the protective effects of IL-22 on aortic dissection with acute lung injury. Dis. Markers 2017, 1917804. 10.1155/2017/1917804 28827891 PMC5554575

[B27] Report on cardiovascular Health and diseases burden in China:an updated summary of 2020. Chin. Circulation J., 2021, 36(6): 521–545.

[B28] Ritchie M. E. Phipson B. Wu D. Hu Y. Law C. W. Shi W. (2015). Limma powers differential expression analyses for RNA-sequencing and microarray studies. Nucleic Acids Res. 43 (7), e47. 10.1093/nar/gkv007 25605792 PMC4402510

[B29] Rouhi L. Fan S. Cheedipudi S. M. Braza-Boïls A. Molina M. S. Yao Y. (2022). The EP300/TP53 pathway, a suppressor of the Hippo and canonical WNT pathways, is activated in human hearts with arrhythmogenic cardiomyopathy in the absence of overt heart failure. Cardiovasc. Res. 118 (6), 1466–1478. 10.1093/cvr/cvab197 34132777 PMC9074970

[B30] Shoffner A. Cigliola V. Lee N. Ou J. Poss K. D. (2020). Tp53 suppression promotes cardiomyocyte proliferation during zebrafish heart regeneration. Cell Rep. 32 (9), 108089. 10.1016/j.celrep.2020.108089 32877671 PMC7494019

[B31] Szklarczyk D. Gable A. L. Nastou K. C. Lyon D. Kirsch R. Pyysalo S. (2021). The STRING database in 2021: Customizable protein-protein networks, and functional characterization of user-uploaded gene/measurement sets. Nucleic Acids Res. 49 (D1), D605–d612. 10.1093/nar/gkaa1074 33237311 PMC7779004

[B32] Tan X. Chen Y. Li J. Li X. Miao Z. Xin N. (2015). The inhibition of Cdk5 activity after hypoxia/ischemia injury reduces infarct size and promotes functional recovery in neonatal rats. Neuroscience 290, 552–560. 10.1016/j.neuroscience.2015.01.054 25665755

[B33] The gene Ontology resource: 20 years and still GOing strong. Nucleic Acids Res., 2019, 47(D1): D330-d338. 10.1093/nar/gky1055 30395331 PMC6323945

[B34] Wang Y. Tan X. Gao H. Yuan H. Hu R. Jia L. (2018). Magnitude of soluble ST2 as a novel biomarker for acute aortic dissection. Circulation 137 (3), 259–269. 10.1161/CIRCULATIONAHA.117.030469 29146682

[B35] Wu G. Cai J. Han Y. Chen J. Huang Z. P. Chen C. (2014). LincRNA-p21 regulates neointima formation, vascular smooth muscle cell proliferation, apoptosis, and atherosclerosis by enhancing p53 activity. Circulation 130 (17), 1452–1465. 10.1161/CIRCULATIONAHA.114.011675 25156994 PMC4244705

[B36] Yan M. S. Marsden P. A. (2015). Epigenetics in the vascular endothelium: Looking from a different perspective in the epigenomics era. Arterioscler. Thromb. Vasc. Biol. 35 (11), 2297–2306. 10.1161/ATVBAHA.115.305043 26404488 PMC4618795

[B37] Yu G. Wang L. G. Han Y. He Q. Y. (2012). clusterProfiler: an R package for comparing biological themes among gene clusters. Omics 16 (5), 284–287. 10.1089/omi.2011.0118 22455463 PMC3339379

[B38] Zarzour A. Kim H. W. Weintraub N. L. (2019). Epigenetic regulation of vascular diseases. Arterioscler. Thromb. Vasc. Biol. 39 (6), 984–990. 10.1161/ATVBAHA.119.312193 31070469 PMC6531339

[B39] Zhang H. T. Wang X. Z. Zhang Q. M. Zhao H. (2021). Neuroprotection of chromobox 7 knockout in the mouse after cerebral ischemia-reperfusion injury via nuclear factor E2-related factor 2/hemeoxygenase-1 signaling pathway. Hum. Exp. Toxicol. 40 (12), S178–s186. 10.1177/09603271211036122 34353139

[B40] Zhang Y. Wei X. Cao C. Yu F. Li W. Zhao G. (2021). Identifying discriminative features for diagnosis of Kashin-Beck disease among adolescents. BMC Musculoskelet. Disord. 22 (1), 801. 10.1186/s12891-021-04514-z 34537022 PMC8449456

[B41] Zhang Y. Zhang J. J. Liu X. H. Wang L. (2020). CBX7 suppression prevents ischemia-reperfusion injury-induced endoplasmic reticulum stress through the Nrf-2/HO-1 pathway. Am. J. Physiol. Ren. Physiol. 318 (6), F1531–f1538. 10.1152/ajprenal.00088.2020 32390514

[B42] Zhou Y. Yin T. Shi M. Chen M. Wu X. Wang K. (2021). Nobiletin attenuates pathological cardiac remodeling after myocardial infarction via activating PPARγ and PGC1α. PPAR Res. 2021, 9947656. 10.1155/2021/9947656 34422028 PMC8373512

