# Supplementary material for: Identification and validation of differentially expressed chromatin regulators for diagnosis of aortic dissection using integrated bioinformatics analysis and machine-learning algorithms
Source: Front Genet. 2022 Aug 11;13:950613. doi: 10.3389/fgene.2022.950613 (PMC9403720; doi:10.3389/fgene.2022.950613)
Supplement: Supplementary file 1 [file DataSheet1.docx]

**Supplementary Material**

**Supplementary Table S1.** The basic characteristics of recruited participants.

| Characteristics | Control | Aortic dissection | *P* value |
| --- | --- | --- | --- |
|  | N=6 | N=6 |  |
| Age, years | 55.33±9.62 | 54.67±12.48 | 0.9195 |
| Gender, n (%) |  |  | 1 |
| Male | 4(66.67) | 3(50.00) |  |
| Female | 2(33.33) | 3(50.00) |  |
| Systolic blood pressure, mmHg | 132.00±16.59 | 199.33±9.09 | 0.0000 |
| Diastolic blood pressure, mmHg | 83.83±7.83 | 112.33±8.78 | 0.0001 |
| Smoking, n (%) | 2(33.33) | 0(0) | 0.4545 |
| Congenital heart disease, n (%) | 0(0) | 1(16.67) | 1 |
| Diabetes, n (%) | 0(0) | 0(0) | 1 |
| Hyperlipidemia, n (%) | 0(0) | 0(0) | 1 |
| Connective tissue disease, n (%) | 0(0) | 0(0) | 1 |
| Injury, n (%) | 0(0) | 0(0) | 1 |
| Syphilis, n (%) | 0(0) | 0(0) | 1 |

**Supplementary Table S2**. Primers used in this study.

| Gene | Forward Primer | Reverse Primer |
| --- | --- | --- |
| homo-CDK5 | TGTCCAGCGTATCTCAGCA | CCACTGTCTCACCCCTCTC |
| homo-CBX7 | CGACATCACCGCCAACT | GAAGTCCCACCCCAAGC |
| homo-JAK2 | AAAACGATCAAACCCCACT | TACCTTATTCGCTTCCTTGTC |
| homo-TP53 | CCAGATGAAGCTCCCAGA | GGGAAGGGACAGAAGATGA |
| homo-β-actin | TCTCCCAAGTCCACACAGG | GGCACGAAGGCTCATCA |

**Supplementary Table S3.** The complete list of DECRs between AD and control.

| Gene | logFC | P.Value | adj.P.Val |
| --- | --- | --- | --- |
| TOP2A | 1.488532838 | 0.025700084 | 0.275865031 |
| AURKB | 1.08314103 | 0.040940117 | 0.300071214 |
| BUB1 | 0.937798816 | 0.008196254 | 0.248595249 |
| NEK6 | 0.79111901 | 0.009288603 | 0.248595249 |
| SNAI2 | 0.773944775 | 0.02987762 | 0.275865031 |
| GADD45B | 0.773683074 | 0.033042658 | 0.280134819 |
| CDK5 | 0.772862228 | 0.021400953 | 0.275865031 |
| PKM | 0.75466594 | 0.003329848 | 0.243574342 |
| MTA2 | 0.731044646 | 0.036935607 | 0.300071214 |
| INO80B | 0.714374832 | 0.000242223 | 0.189089842 |
| AICDA | 0.65893753 | 0.006844391 | 0.248595249 |
| CHEK1 | 0.655668565 | 0.025182064 | 0.275865031 |
| MAPKAPK3 | 0.649684725 | 0.026109823 | 0.275865031 |
| TP53 | 0.615045373 | 0.000467134 | 0.189089842 |
| ZBTB16 | -0.596380009 | 0.026045511 | 0.275865031 |
| TDRD5 | -0.606053424 | 0.028453014 | 0.275865031 |
| PCGF5 | -0.606512712 | 0.009053092 | 0.248595249 |
| ZBTB7C | -0.61848015 | 0.031916639 | 0.280134819 |
| KDM3A | -0.641237256 | 0.008657377 | 0.248595249 |
| TOX2 | -0.641724436 | 0.047974024 | 0.317415572 |
| CBX7 | -0.659397776 | 0.032139191 | 0.280134819 |
| JAK2 | -0.729308143 | 0.012836503 | 0.253845074 |
| TRDMT1 | -0.815838465 | 0.000899357 | 0.189089842 |

Abbreviations: DECRs, differentially expressed chromatin regulators; AD, aortic dissection; FC, fold change; adj.P.Val, adjusted P value.

**Supplementary Table S4.** Functional enrichment analysis of DECRs in AD.

| ID | Description | p.adjust | Count | Gene |
| --- | --- | --- | --- | --- |
| **Biological Process** | |  |  |  |
| GO:0043412 | macromolecule modification | 7.022E-06 | 18 | INO80B/TP53/TRDMT1/AICDA/BUB1/  KDM3A/PCGF5/NEK6/JAK2/CDK5/CHEK1/  ZBTB16/MAPKAPK3/TDRD5/SNAI2/  GADD45B/MTA2/AURKB |
| GO:0051173 | positive regulation of nitrogen compound metabolic process | 5.65E-06 | 16 | TP53/AICDA/KDM3A/PCGF5/JAK2/CDK5/  CHEK1/TOP2A/ZBTB16/MAPKAPK3/SNAI2/  ZBTB7C/GADD45B/MTA2/AURKB/TOX2 |
| GO:0031325 | positive regulation of cellular metabolic process | 7.375E-06 | 16 | TP53/AICDA/KDM3A/PCGF5/JAK2/CDK5/  CHEK1/TOP2A/ZBTB16/MAPKAPK3/SNAI2/  ZBTB7C/GADD45B/MTA2/AURKB/TOX2 |
| GO:0010604 | positive regulation of macromolecule metabolic process | 7.375E-06 | 16 | TP53/AICDA/KDM3A/PCGF5/JAK2/CDK5/  CHEK1/TOP2A/ZBTB16/MAPKAPK3/SNAI2/  ZBTB7C/GADD45B/MTA2/AURKB/TOX2 |
| GO:0009893 | positive regulation of metabolic process | 2.115E-05 | 16 | TP53/AICDA/KDM3A/PCGF5/JAK2/CDK5/  CHEK1/TOP2A/ZBTB16/MAPKAPK3/SNAI2/  ZBTB7C/GADD45B/MTA2/AURKB/TOX2 |
| GO:0006996 | organelle organization | 2.314E-05 | 16 | INO80B/TP53/AICDA/BUB1/KDM3A/PCGF5/  NEK6/JAK2/CDK5/CHEK1/TOP2A/TDRD5/  SNAI2/CBX7/MTA2/AURKB |
| GO:0034654 | nucleobase-containing compound biosynthetic process | 0.000155 | 16 | TP53/PKM/AICDA/KDM3A/PCGF5/JAK2/  CDK5/CHEK1/TOP2A/ZBTB16/SNAI2/  ZBTB7C/CBX7/MTA2/AURKB/TOX2 |
| GO:0018130 | heterocycle biosynthetic process | 0.0001705 | 16 | TP53/PKM/AICDA/KDM3A/PCGF5/JAK2/  CDK5/CHEK1/TOP2A/ZBTB16/SNAI2/  ZBTB7C/CBX7/MTA2/AURKB/TOX2 |
| GO:0019438 | aromatic compound biosynthetic process | 0.0001705 | 16 | TP53/PKM/AICDA/KDM3A/PCGF5/JAK2/  CDK5/CHEK1/TOP2A/ZBTB16/SNAI2/  ZBTB7C/CBX7/MTA2/AURKB/TOX2 |
| GO:1901362 | organic cyclic compound biosynthetic process | 0.0002334 | 16 | TP53/PKM/AICDA/KDM3A/PCGF5/JAK2/  CDK5/CHEK1/TOP2A/ZBTB16/SNAI2/  ZBTB7C/CBX7/MTA2/AURKB/TOX2 |
| **Cellular Component** | |  |  |  |
| GO:0005654 | nucleoplasm | 1.238E-07 | 18 | INO80B/TP53/TRDMT1/BUB1/KDM3A/  PCGF5/NEK6/JAK2/CDK5/CHEK1/TOP2A/  ZBTB16/MAPKAPK3/SNAI2/CBX7/MTA2/  AURKB/TOX2 |
| GO:0031981 | nuclear lumen | 3.373E-06 | 18 | INO80B/TP53/TRDMT1/BUB1/KDM3A/  PCGF5/NEK6/JAK2/CDK5/CHEK1/TOP2A/  ZBTB16/MAPKAPK3/SNAI2/CBX7/MTA2/  AURKB/TOX2 |
| GO:0044428 | nuclear part | 6.983E-06 | 18 | INO80B/TP53/TRDMT1/BUB1/KDM3A/  PCGF5/NEK6/JAK2/CDK5/CHEK1/TOP2A/  ZBTB16/MAPKAPK3/SNAI2/CBX7/MTA2/  AURKB/TOX2 |
| GO:0032991 | protein-containing complex | 0.0017806 | 15 | INO80B/TP53/PKM/AICDA/BUB1/PCGF5/  NEK6/CDK5/CHEK1/TOP2A/ZBTB16/  TDRD5/CBX7/MTA2/AURKB |
| GO:0043232 | intracellular non-membrane-bounded organelle | 0.0021769 | 14 | INO80B/TP53/BUB1/PCGF5/NEK6/JAK2/  CDK5/CHEK1/TOP2A/TDRD5/SNAI2/  CBX7/MTA2/AURKB |
| GO:0043228 | non-membrane-bounded organelle | 0.0021769 | 14 | INO80B/TP53/BUB1/PCGF5/NEK6/JAK2/  CDK5/CHEK1/TOP2A/TDRD5/SNAI2/  CBX7/MTA2/AURKB |
| GO:0005829 | cytosol | 0.0719451 | 11 | TP53/PKM/BUB1/NEK6/JAK2/CDK5/  CHEK1/ZBTB16/MAPKAPK3/CBX7/  AURKB |
| GO:0005694 | chromosome | 4.176E-06 | 10 | INO80B/TP53/BUB1/PCGF5/CHEK1/  TOP2A/SNAI2/CBX7/MTA2/AURKB |
| GO:0000228 | nuclear chromosome | 3.848E-07 | 9 | INO80B/TP53/BUB1/CHEK1/TOP2A/  SNAI2/CBX7/MTA2/AURKB |
| GO:0044427 | chromosomal part | 0.0001254 | 8 | INO80B/TP53/BUB1/CHEK1/SNAI2/  CBX7/MTA2/AURKB |
| **Molecular Function** | |  |  |  |
| GO:0046872 | metal ion binding | 0.001132 | 14 | INO80B/TP53/PKM/AICDA/KDM3A/  PCGF5/NEK6/JAK2/TOP2A/ZBTB16/  SNAI2/ZBTB7C/MTA2/AURKB |
| GO:0043169 | cation binding | 0.0012451 | 14 | INO80B/TP53/PKM/AICDA/KDM3A/  PCGF5/NEK6/JAK2/TOP2A/ZBTB16/  SNAI2/ZBTB7C/MTA2/AURKB |
| GO:0003824 | catalytic activity | 0.013694 | 13 | TRDMT1/PKM/AICDA/BUB1/KDM3A/  NEK6/JAK2/CDK5/CHEK1/TOP2A/  MAPKAPK3/MTA2/AURKB |
| GO:0005524 | ATP binding | 0.0001694 | 10 | TP53/PKM/BUB1/NEK6/JAK2/CDK5/  CHEK1/TOP2A/MAPKAPK3/AURKB |
| GO:0032559 | adenyl ribonucleotide binding | 0.0001694 | 10 | TP53/PKM/BUB1/NEK6/JAK2/CDK5/  CHEK1/TOP2A/MAPKAPK3/AURKB |
| GO:0030554 | adenyl nucleotide binding | 0.0001694 | 10 | TP53/PKM/BUB1/NEK6/JAK2/CDK5/  CHEK1/TOP2A/MAPKAPK3/AURKB |
| GO:0008144 | drug binding | 0.0003534 | 10 | TP53/PKM/BUB1/NEK6/JAK2/CDK5/  CHEK1/TOP2A/MAPKAPK3/AURKB |
| GO:0035639 | purine ribonucleoside triphosphate binding | 0.0004727 | 10 | TP53/PKM/BUB1/NEK6/JAK2/CDK5/  CHEK1/TOP2A/MAPKAPK3/AURKB |
| GO:0032555 | purine ribonucleotide binding | 0.0005311 | 10 | TP53/PKM/BUB1/NEK6/JAK2/CDK5/  CHEK1/TOP2A/MAPKAPK3/AURKB |
| GO:0017076 | purine nucleotide binding | 0.0005311 | 10 | TP53/PKM/BUB1/NEK6/JAK2/CDK5/  CHEK1/TOP2A/MAPKAPK3/AURKB |
| **KEGG** |  |  |  |  |
| hsa04110 | Cell cycle | 0.0044283 | 4 | TP53/BUB1/CHEK1/GADD45B |
| hsa05200 | Pathways in cancer | 0.0560703 | 4 | TP53/JAK2/ZBTB16/GADD45B |
| hsa04115 | p53 signaling pathway | 0.0106404 | 3 | TP53/CHEK1/GADD45B |
| hsa04218 | Cellular senescence | 0.0445795 | 3 | TP53/CHEK1/GADD45B |
| hsa05202 | Transcriptional mis-regulation in cancer | 0.0445795 | 3 | TP53/ZBTB16/GADD45B |
| hsa05203 | Viral carcinogenesis | 0.0445795 | 3 | TP53/PKM/CHEK1 |
| hsa04010 | MAPK signaling pathway | 0.0629529 | 3 | TP53/MAPKAPK3/GADD45B |
| hsa05216 | Thyroid cancer | 0.0445795 | 2 | TP53/GADD45B |
| hsa05213 | Endometrial cancer | 0.0445795 | 2 | TP53/GADD45B |
| hsa05217 | Basal cell carcinoma | 0.0445795 | 2 | TP53/GADD45B |

Abbreviations: DECRs, differentially expressed chromatin regulators; AD, aortic dissection; KEGG, Kyoto Encyclopedia of Genes and Genomes.
